# Sept heures pour une désensibilisation efficace et sans risque chez les patients VIH positifs intolérants au cotrimoxazole

**DOI:** 10.11604/pamj.2014.18.238.4820

**Published:** 2014-07-22

**Authors:** Jalal El Benaye, Bekkali Nihal, Janah Hicham, Elhaouri Mohamed

**Affiliations:** 1 Service de Dermatologie, Hôpital Militaire d'Instruction Mohammed V, Rabat, Maroc; 2 Service de Pneumologie, Hôpital Militaire d'Instruction Mohammed V, Rabat, Maroc; 3 Service de Dermatologie, Hôpital Militaire Moulay Ismail, Meknès, Maroc

**Keywords:** Désensibilisation, VIH positif, intolérant, cotrimoxazole, desensitization, HIV positif, intolerant, cotrimoxazole

## Aux editeurs du Journal Panafricain de Médecine

Chez les sujets VIH positifs, le cotrimoxazole, largement utilisé en prophylaxie et/ou dans le traitement de certaines infections opportunistes, est responsable de la majeure partie des toxidermies. En l'absence d'alternative thérapeutique aussi efficace et disponible que moins chère, la désensibilisation reste un atout stratégique intéressant, efficace et sans danger. Nous en rapportons 5 observations ayant bénéficié d'un protocole de désensibilisation de 7 heures. Le cotrimoxazole était prescrit dans 2 cas de pneumocystose pulmonaire et dans 3 cas comme antibioprophylaxie. L'intolérance consistait en un rash maculo-papuleux prurigineux sans décollement ni atteinte muqueuse ni systémique. Un mois après la régression de l'intolérance, une réintroduction du cotrimoxazole s'est faite en milieu hospitalier sous surveillance étroite et consistait en l'administration de doses progressives du cotrimoxazole sirop pédiatrique, doublant la dose chaque heure jusqu’à dose cible 400mg/80mg, selon le protocole rapporté dans le Tableau 1 et de prendre la pleine dose à partir du lendemain. Ce protocole devait être interrompu immédiatement si un signe de gravité apparaissait, chose qui ne s'est pas produite et nos patients continuaient à tolérer le cotrimoxazole.

**Tableau 1:** Protocole de désensibilisation en 7 heures

Heures	Dose (ml)	Dose (mg)	Observations
8H	0,1	4/0,8	Pas de réaction
9H	0,2	8/1,6	
10H	0,4	16/3,2	
11H	0,8	32/6,4	
12H	1,6	64/12,8	
13H	3,2	128/25,6	
14H	6,4	256/51,2	
15H	10	400/80	

La désensibilisation se définit par le passage d'un état d'hypersensibilité à un état de tolérance par l'introduction prudente de quantités croissantes d′antigène à intervalles de temps réguliers. Son efficacité dans l'hypersensibilité au cotrimoxazole n'est plus contestée et serait de l'ordre de 60 à 100% [1]. Néanmoins, son mécanisme ainsi que celui de l'intolérance au cotrimoxazole dans le cadre du VIH, restent à éclaircir. Cette dernière serait multifactorielle: génétique, métabolique, toxique et/ou immunologique [2]. Plusieurs protocoles de désensibilisation sont utilisés, tous avec une efficacité similaire. Certains travaux ont suggéré que les protocoles étalés sur plusieurs jours seraient plus efficaces que les protocoles courts [3], notion non partagée par la majorité des auteurs qui ont démontré que les protocoles courts, y compris celui en un jour, peuvent être aussi efficace à court et à long terme même chez l'enfant [4]. Bonfanti et al ont comparé la désensibilisation et la réintroduction pleine-dose du cotrimoxazole et ont conclu que les deux méthodes avaient la même efficacité [5]. Cependant, les patients ayant bénéficié d'une réintroduction pleine-dose souffriraient plus de fièvre et de céphalées [2]. La désensibilisation serait préférée à la réintroduction pleine-dose de part son efficacité qui reste supérieure et de part sa sécurité [1].

Notre expérience rejoint les données de la littérature en ce qui concerne l'efficacité et la sécurité de la désensibilisation notamment en un jour, ce qui permettrait une meilleure surveillance et observance thérapeutique et réduirait le coût des hospitalisations.

## Conflits d’‘intérêts

Les auteurs ne déclarent aucun conflits d’‘intérêts
